# Abnormal anti-Müllerian hormone level may be a trigger for breast cancer in young women: A case-control study

**DOI:** 10.18502/ijrm.v19i2.8476

**Published:** 2021-02-21

**Authors:** Amirmohsen Jalaeefar, Ashraf Moini, Bita Eslami, Sadaf Alipour, Mohammad Shirkhoda, Arvin Aryan, Habibollah Mahmoodzadeh, Ramesh Omranipour

**Affiliations:** ^1^Department of Surgical Oncology, Cancer Institute, Tehran University of Medical Sciences, Tehran, Iran.; ^2^Breast Disease Research Center (BDRC), Tehran University of Medical Sciences, Tehran, Iran.; ^3^Department of Gynecology and Obstetrics, Arash Women's Hospital, Tehran University of Medical Sciences, Tehran, Iran.; ^4^Department of Endocrinology and Female Infertility at Reproductive Biomedicine Research Center, Royan Institute for Reproductive Biomedicine, ACECR, Tehran, Iran.; ^5^Department of Surgery, Arash Women's Hospital, Tehran University of Medical Sciences, Tehran, Iran.; ^6^Department of Radiology, Advanced Diagnostic and Interventional Radiology Research Center (ADIR), Imam Khomeini Hospital, Tehran University of Medical Sciences, Tehran, Iran.

**Keywords:** Anti-Müllerian hormone, Breast cancer, Biomarkers, Ovarian reserve.

## Abstract

**Background:**

Anti-Müllerian hormone (AMH) is a known sensitive biomarker for fertility and ovarian reserve. The results of in vivo and human studies showed inconsistency with respect to the relation between AMH and breast cancer.

**Objective:**

To compare the AMH level of young Iranian women with early breast cancer who have not received any treatment compared to that of healthy women.

**Materials and Methods:**

In this case-control study, 58 breast cancer cases were recruited from the breast oncology clinic of two university hospitals. They were diagnosed with an in situ or invasive breast cancer before any anticancer treatment between August 2018 and April 2019. Healthy controls (n = 58) were selected from women referred to a gynecologic outpatient clinic without any symptoms of cancer or infertility. AMH was measured by the AMH enzyme-linked immunosorbent assay kits in one laboratory.

**Results:**

Final analysis showed that the AMH means of case and control were not statistically significant (3.36 ± 2.95 vs 3.13 ± 1.79). However, the lower and higher AMH level categories are more prevalent in breast cancer compared to the control. Pearson's correlation test showed that the AMH level was negatively correlated with age (r = -0.44, p< 0.001). The results of logistic regression analysis considering confounding factors showed the positive association between breast cancer and lower (Odds Ratio [OR] = 5.98, p = 0.02) and higher quartile of AMH level (OR = 4.95, p = 0.01).

**Conclusion:**

Our results suggest that abnormal AMH level is more frequent in young breast cancer patients. Further investigation considering AMH determinants is required.

## 1. Introduction

Breast cancer is the most commonly diagnosed cancer in most regions of the world (1). Approximately 9.5% of new cases of breast cancer and 6.7% of breast cancer occur among women aged < 45 yr (2). A recent study has shown that the incidence of breast cancer in young women is increasing (3); the mean age of the women with breast cancer in Iran is < 50 yr (4).

Scientists are looking for different biomarkers to prevent or treat cancers including breast cancer. One of the researchers' favorite biomarkers is Müllerian-inhibiting substance, also known as anti-Müllerian hormone (AMH). Besides the use of AMH as a sensitive biomarker for fertility and ovarian reserve, it serves as a biomarker in several other conditions (5). For instance, AMH concentration is used as a reliable marker if at any time there are minor fluctuations in serum concentrations during a normal menstrual cycle (6).

AMH is a member of the transforming growth factor-β superfamily of the growth and differentiation response modifiers. Because most gynecologic tumors originate from Müllerian duct-derived tissues, and since Müllerian-inhibiting substance/AMH causes regression of the Müllerian duct in male embryos, it is expected to have an inhibiting effect on the growth of gynecologic tumors (7).

Based on laboratory evidence in cell-line and mouse models, an inverse association between AMH level and breast tumor development has been illustrated (8-11), and it seems that AMH has a protective effect against breast cancer and can suppress the growth of mammary tumors. These findings have led to the suggestion that AMH could be used in the treatment of breast and other gynecologic cancers (7). Although the exact role of AMH in mammary glands is still unclear, they are possible target tissue of AMH.

However, different results have been reported in epidemiological human studies. Some of them assert that a lower AMH is associated with a higher rate of breast cancers (12, 13), while others have shown a positive relationship between AMH level and breast cancer (14-17). Since AMH plays a role in regulating folliculogenesis and the interaction between AMH and estradiol is not fully understood (18), the abnormal AMH level may affect estradiol concentration that will cause breast cancer. A question that still needs to be investigated is whether the AMH level before any anticancer treatment is impacted by cancer itself or abnormal AMH level is one of the risk factors of breast cancer?

Because of the inconsistent reported results about the association between AMH level and breast cancer, our aim in the present study was to measure the level of AMH in young Iranian breast cancer patients and compare it with healthy controls.

## 2. Materials and Methods

### Study design and participants selection

This case-control study was conducted at two University Hospitals (Cancer Institute and Arash Women's Hospitals) of Tehran University of Medical Sciences, Tehran, Iran, between August 2018 and April 2019.

Fifty-eight women who recently received a biopsy-proven diagnosis of breast cancer, attending the breast oncology clinic as the case group. Furthermore, they had not received any anticancer treatment. In the control group (n = 58) consisted of women attending the Same Hospitals' Gynecologic Outpatient Clinic for routine check-ups and those who had not reported a previous or current symptoms of cancer or infertility.

The inclusion criteria for both groups were: age < 40 yr and regular menstrual cycles as a defined absence of menopause. The exclusion criteria were: age > 40, irregular menstrual cycles, a previous or current history of infertility, and any symptoms of menopause.

A trained interviewer gathered individual information about age, height, weight, education, marital status, reproductive history, occupation, history of a disease, and oral contraceptive use through in person-interview. To evaluate the antral follicle count (AFC), we offered transvaginal sonography for all participants if they agreed. Immunohistochemistry (IHC) was performed on all breast cancer specimens and results were recorded as estrogen receptor (ER), progesterone receptor (PR), and human epidermal growth factor-2 (HER2) status.

Body mass index (BMI) was calculated by the formula of weight divided by height squared (kg/m${}^{2}$) and categorized based on the WHO classification (19).

### AMH measurement

Hospital nurses collected 5 ml of blood samples from the cubital vein. All samples were stored at room temperature and transferred to the same laboratory within 2 hr. The laboratory doctor and personnel were blinded about case and control groupings.

AMH was measured by the AMH enzyme-linked immunosorbent assay kits (Beckman Coulter, AMH gene II assay, Brea, CA, USA). The limit of detection (LOD) was 0.08 ng/ml intra-assay and inter-assay variations were 7.7%.

### Sample size calculation

Based on the results of a study (14) and considering the OR of higher quartile compared to the reference quartile, we calculated that at least 44 samples would be required in each group to detect an association with a power of 80% and α = 0.05 by using Epi Info site (http:www.cdc.gov/epiinfo). In each group, 60 samples were recruited for possible losses and further analysis.

### Ethical considerations

This study was approved by the ethics committee of Tehran University of Medical Sciences (IR.TUMS.VCR.REC.1397.693). Appropriate written informed consent was obtained from all participants prior to blood sample collection.

### Statistical analysis

Data were analyzed using the SPSS software (version 20, SPSS, Inc., IL, USA). Results are presented as Mean ± Standard Deviation (SD) for continuous variables and frequency for categorical variables. The differences in means were tested by student's *t* test or ANOVA test. Categorical variables differences were tested by the Chi-square test (χ${}^{2}$). A two-sided p-value < 0.05 was considered as significant.

Multivariable binary logistic regression (backward stepwise method) was used to estimate adjusted OR and 95% confidence interval (CI) for assessing the association between breast cancer and AMH level. Final statistical model accounted for age (continuous), age at menarche (continuous), parity (nulliparous/multiparous), breastfeeding duration (continuous), and BMI (< 18.5, 18.5-24.9, 25-29.9, ≥ 30 kg/m${}^{2}$) as potential confounders and AMH quartiles (< 1.41, 1.41-2.66, 2.67-4.45, > 4.45 ng/mL). The second and third quartiles of the AMH level (1.41-4.45 ng/mL) were considered as a reference value.

## 3. Results

The final analysis was performed on the data collected from 58 women in each group. Table I presents the Characteristics of cases and controls. Overall, breast cancer patients had lower single status women compared to the control group, and women in the control group were more educated than those in the case group with borderline significance (p = 0.07). With respect to the medical history of the participants, thyroid diseases were common and other diseases were very rare. Therefore, self-history of thyroid diseases is mentioned in Table I, which was more common in the breast cancer group compared to the control (12% vs 3.4%, p = 0.08).

AMH was detected in all samples. The distribution of AMH level was marginally normal (Kolmogrove-Sminrnove test, p = 0.05). Table II shows the descriptive value and categorical AMH levels in both groups. Pearson's correlation test showed that the AMH level was negatively correlated with age (r = -0.45, p < 0.001) (Data not presented in the table). A comparisons of the AMH levels between the two groups showed that the mean concentration of AMH was not significantly higher in the case group (p = 0.45).

However, a comparison of AMH quartiles between the two groups showed that the number of cases belonging to the first and fourth quartiles was significantly higher than the controls (p < 0.001). AFC in each ovary was evaluated in 57 and 24 of the controls and cases, respectively, and the average number of antral follicles in ovaries was similar in both groups (Right: 6.70 ± 3.33 vs 6.71 ± 3.42, p = 0.99; Left: 6.86 ± 3.70 vs 6.54 ± 3.74, p = 0.73). Table III shows that the AMH level decreased significantly by age increase.

AMH level was significantly higher in HER2-negative compared with HER2-positive (p = 0.02) cancers. Meanwhile, AMH level was higher in ER-positive compared to ER-negative, but not statistically significant (p = 0.07).

The adjusted ORs for breast cancer in relation to AMH quartiles are shown in table IV. In univariate analysis, there was a statistically significant increasing risk with lower and higher quartiles of AMH concentration. Results were similar after adjustment for potential confounders.

**Table 1:** Total characteristics of cases and controls


**Variables**		**Breast cancer (n = 58)**	**Control (n = 58)**	**P-value**
Age (yr)*		34.88 ± 4.23	34.10 ± 3.28	0.28
BMI (kg/m${}^{2}$)*		25.21 ± 4.24	25.79 ± 4.72	0.49
Age of menarche*		13.39 ± 1.28	13.10 ± 1.17	0.22
Age at first pregnancy*		23.68 ± 5.16	24.46 ± 4.80	0.49
Parity (n)*		1.61 ± 0.88	1.43 ± 0.60	0.30
Lactation period (mo)*		24.02 ± 22.46	16.72 ± 2.63	0.07
Education**				
	**Graduate**	24 (41.4)	23 (39.7)	0.07
	**Diploma**	14 (24.1)	25 (43.1)	
	**6-11 yr**	7 (12.1)	5 (8.6)	
	**< 6 yr**	13 (22.4)	5 (8.6)	
Marital status**				
	**Single**	12 (20.7)	21 (36.2)	0.049
	**Married**	46 (79.3)	37 (63.8)	
**Job**				
	**Housewife**	43 (74.1)	44 (75.9)	0.97
	**Office worker**	10 (17.3)	9 (15.5)	
	**Others**	5 (8.6)	5 (8.6)	
History of abortion**		17 (29.3)	11 (19)	0.28
OCP use**		19 (32.8)	22 (37.9)	0.70
History of thyroid disease **		7 (12)	2 (3.4)	0.08
PCOS**		5 (8.6)	9 (15.5)	0.39
BMI category**				
	**< 18.5**	1 (1.7)	1 (1.7)	0.95
	**18.5-24.99**	27 (46.5)	29 (50)	
	**25-29.99**	23 (39.7)	20 (34.5)	
	**≥ 30**	7 (12.1)	8 (13.8)	
*Data presented as Mean ± standard deviation. **Data presented as n (%). Continuous variables were compared using the student's *t* test, while categorical variables were examined using Chi-square test. BMI: Body mass index, OCP: Oral contraceptive pill, PCOS: Polycystic ovarian syndrome				

**Table 2:** AMH level and categories in both groups


	**Breast cancer (n = 58)**	**Control (n = 58)**	**P-value**
**AMH Level**				
	Arithmetic *	3.36 ± 2.95	3.13 ± 1.79	0.45
	**Median**	2.48	2.75	
	**Min-max**	0.08-10.60	0.2-9.60	
AMH category (quartile)**				
	**< 1.41**	21 (36.2)	8 (13.8)	0.001			
	**1.41-2.66**	10 (17.2)	19 (32.8)
	**2.67-4.45**	8 (13.8)	21 (36.2)
	**> 4.45**	19 (32.8)	10 (17.2)
*Data presented as Mean ± SD. **Data presented as n (%). AMH mean level was compared using the student's *t* test and AMH categories were compared using the Chi-square test. AMH: Anti-Müllerian hormone, SD: Standard deviation, Ln: Natural logarithm				

**Table 3:** Determinants of AMH level in the studied samples


**Variable**		**N**	**Mean ± SD**	**P-value**
**Age quartiles (yr)**				
	**< 31**	30	4.76 ± 2.73	< 0.001*
	**32-35**	37	3.60 ± 2.31	
	**36-38**	27	2.19 ± 1.57	
	**> 38**	22	1.88 ± 1.69	
**BMI (kg/m${}^{2}$)**				
	**< 18.5**	2	1.85 ± 0.63	0.17*
	**18.5-24.99**	56	3.16 ± 2.32	
	**25-29.99**	43	3.76 ± 2.71	
	**≥ 30**	15	2.29 ± 1.73	
**Lactation period quartile (mo)**				
	**No lactation**	45	2.92 ± 1.99	0.43*
	**1-17 **	10	4.26 ± 2.93	
	**18-38**	32	3.19 ± 2.12	
	**≥ 39**	29	3.46 ± 3.10	
**Parity **				
	**Nulliparity**	36	2.93 ± 2.17	0.35**
	**Multiparity**	80	3.39 ± 2.53	
**History of abortion **				
	**No**	88	3.14 ± 2.30	0.39**
	**Yes**	28	3.59 ± 2.80	
**PCOS **				
	**No**	102	3.18 ± 2.47	0.40**
	**Yes**	14	3.76 ± 2.10	
**OCP use **				
	**No**	75	2.99 ± 2.13	0.15**
	**Yes**	41	3.72 ± 2.86	
**ER **				
	**Positive**	26	3.63 ± 3.05	0.07**
	**Negative**	14	2.20 ± 1.81	
**HER-2 **				
	**Positive**	12	1.74 ± 2.20	0.03**
	**Negative**	28	3.73 ± 2.77	
	**Triple-negative**	9	2.34 ± 1.42	-
*P-value refers to the ANOVA-test. **P-value refers to the student's *t* test. SD: Standard deviation, BMI: Body mass index, PCOS: Polycystic ovarian syndrome, OCP: Oral contraceptive pills, ER: Estrogen receptor, HER2: Human epidermal growth factor receptor 2				

**Table 4:** Unadjusted and adjusted odds ratio from logistic regression for AMH level associated with breast cancer


**Variables**	**Cases/controls**	**Unadjusted OR (95% CI)**	**P-value**	**Adjusted OR (95% CI)**	**P-value**
**AMH level**			
**Q2, 3: 1.41-4.45**	18/40	1 (Referent)	1 (Referent)	
**Q1: < 1.41**	21/8	5.83 (2.18-15.64)	< 0.001	5.98 (1.42-25.23)	0.02
**Q4: > 4.45**	19/10	4.22 (1.64-10.88)	0.003	4.95 (1.44-16.98)	0.01
white<bcol>6</ecol>Adjusted OR was calculated considering confounding factors (age, age of menarche, parity, BMI categories, AMH quartiles, lactation period). OR: Odds ratio, AMH: Anti-Müllerian hormone

## 4. Discussion

The serum levels of AMH in young women with early breast cancer prior to any treatment was compared to healthy women in the present study considering confounding factors. We found a positive association between lower and higher AMH levels and breast cancer before anticancer treatment.

Our results were not consistent with preclinical studies in cell-line and animal models. Those studies reported that AMH inhibits tumor cell growth and migration through nuclear factor kappa-light-chain-enhancer of activated B cells-mediated pathways, and that after AMH injection, the ratio of apoptotic cells in murine mammary tissue increases eightfold (8-11). Therefore, an inverse association between AMH and breast tumor development was demonstrated. However, those models have evaluated the basal-like breast cancer subtypes, which account for the minority of breast cancers. As mentioned in Table III, the AMH level in triple-negative patients is this study was 2.34 ± 1.42. It seems that AMH assessment in other subtypes of breast cancer is necessary.

The present study result shows that the low level of AMH in young patients is associated with breast cancer, which has also been confirmed by other studies (12, 13). McCoy and colleagues measured the AMH level in premenopausal women aged 38-50 yr scheduled to undergo diagnostic breast biopsy to determine the relation with benign or malignant breast lesions. They observed a negative association of AMH level with precancerous and cancerous breast diseases. However, the sample size of that study was very low (12). Another epidemiologic study by Su and co-workers on very young breast cancer patients (28-44 yr) found a significantly lower AMH in breast cancer patients in univariate analysis, and AMH levels that were more frequently below the detection rates in breast cancer patients than that of controls. However, in the multivariable model, they didn't find any significant difference in the AMH levels between cancerous patients and healthy women of similar age. They mentioned that ovarian reserve may be adversely impacted by cancer status (13). Another study demonstrated that women with cancer may have significantly lower AFC before gonadotoxic therapy compared with healthy women aged 25-40 yr (20). AMH level is correlated with AFC and this lower AFC and subsequent AMH level in cancer patients may be explained by the accelerated follicle loss or a defect in the recruitment of antral follicles owning to a disease state.

It is well-known that high breast density is associated with an increased risk of breast cancer (21). A recent study examined the relation of AMH with mammographic breast density. They found that women with higher AMH levels had a significantly lower fatty breast (22). This study supported the hypothesis of the relationship between AMH levels and breast cancer. Meanwhile, this study could support the previous findings which explained the possible effect of AMH on breast tissue.

Unlike the aforementioned studies, some investigations show a positive relationship between breast cancer and AMH level (14-17). The first prospective study in premenopausal women reported a strong association of AMH (OR = 9.8, 95% CI: 3.3-28.9) with breast cancer before the diagnosis, although a weaker association was observed in breast cancer patients who were not using oral contraceptive during blood collection (14). They suggested that control subjects of their study were not representative of unaffected women in the cohort and stated that the association between AMH and breast cancer could be biased.

A large-scale study that evaluated the AMH level and risk of breast cancer in 10 prospective cohorts reported a positive relationship between breast cancer and AMH level (17). However, that study sample population consisted of premenopausal women of any age at the time of blood donation. Further, two recent prospective studies observed a strong positive association between increasing categories of AMH and breast cancer. In these two studies, the association was weaker among younger women and neither study reported the menopausal status at the time of diagnosis (15, 16). In Elaissen and colleagues study, control groups were selected without considering menopausal status, and they allowed postmenopausal women among controls; this caused a higher estimation of the association between breast cancer and AMH (16). Since Rustamov and co-workers have shown that AMH level is reproducible over time like other biologic variables (23), it should be considered especially in the cohort study which evaluated the hormone level after many years of sample collection.

Our results showed that the AMH level is higher in the PCOS group (Table III) as we expected based on previous study (24), although it was not statistically significant. Blumenfeld (25) has discussed the recent study by Ge and colleagues which has found that the breast cancer has a positive relation with AMH level (17). Blumenfeld has mentioned that the positive relation of breast cancer with AMH level may be due to a higher prevalence of PCOS patients in the high AMH-level group (25). His argument is based on the possible relationship between breast cancer and PCOS, which of course has been rejected in the recently published systematic review and meta-analysis (26, 27).

According to IHC of breast cancer patients, AMH level was higher in ER-positive (p = 0.07) and Her2-negative tumors (p = 0.03) (Table III). Although our results require further investigation due to the small sample size and lack of access to IHC of all patients, Ge and co-authors study also confirmed the increasing trend of AMH for tumors positive for both estrogen and progesterone receptors (17). It seems that further evaluation of AMH level in subtypes of breast cancer is needed for appropriate fertility preservation counseling.

A recent study showed that breast cancer patients with *BRCA* mutation have significantly lower serum AMH levels and recommended that fertility preservation should be considered more aggressively in these patients (28). Therefore, for the accurate evaluation of the association between AMH level and breast cancer, BRCA mutation assessment is necessary.

The present study has some advantages. Based on our knowledge, this is the first evaluation of the AMH level in young Iranian breast cancer patients. In this study, we measured AMH levels for 2 hours after sampling and used the AMH Gene II assay kit for measurement, which is highly specific and reproducible (29).

##  Limitation

The first limitation of the current study is that since the AMH level was evaluated after a breast cancer diagnosis, it might not reflect the AMH level before cancer development. Another limitation might the small sample size of the study compared with large cohorts.

## 5. Conclusion

In conclusion, we found the lower and higher AMH level categories are more prevalent in young breast cancer patients who were in premenopausal status and didn't receive any cytotoxic treatment. Further studies are necessary with a larger sample size, considering age, menopausal status, oral contraceptive use, and previous history of other diseases such as PCOS as well as genetic assessment for BRCA mutation. As young women are more likely to benefit from preventive drug intervention, individual risk assessment is particularly valuable in young women and can encourage them to pursue screening for early detection of breast cancer, or offer them new biomarker-based treatments.

##  Conflict of Interest

The authors declare that they have no conflict of interest.
